# Cold Atmospheric Plasma Promotes Regeneration-Associated Cell Functions of Murine Cementoblasts In Vitro

**DOI:** 10.3390/ijms22105280

**Published:** 2021-05-17

**Authors:** Benedikt Eggers, Jana Marciniak, James Deschner, Matthias Bernhard Stope, Alexander Mustea, Franz-Josef Kramer, Marjan Nokhbehsaim

**Affiliations:** 1 Department of Oral, Maxillofacial and Plastic Surgery, University Hospital Bonn, 53111 Bonn, Germany; franz-josef.kramer@ukbonn.de; 2 Department of Orthodontics, University Hospital Bonn, 53111 Bonn, Germany; jana.marciniak@ukbonn.de; 3 Department of Periodontology and Operative Dentistry, University Medical Center of the Johannes Gutenberg University, 55131 Mainz, Germany; james.deschner@uni-mainz.de; 4 Department of Gynecology and Gynecological Oncology, University Hospital Bonn, 53111 Bonn, Germany; matthias.stope@ukbonn.de (M.B.S.); alexander.mustea@ukbonn.de (A.M.); 5 Section of Experimental Dento-Maxillo-Facial Medicine, University Hospital Bonn, 53111 Bonn, Germany; m.saim@uni-bonn.de

**Keywords:** cold atmospheric plasma, emdogain, cementoblasts, mineralization, proliferation, traumatic dental injuries, dental hard tissue regeneration therapy

## Abstract

The aim of the study was to examine the efficacy of cold atmospheric plasma (CAP) on the mineralization and cell proliferation of murine dental cementoblasts. Cells were treated with CAP and enamel matrix derivates (EMD). Gene expression of alkaline phosphatase (ALP), bone gamma-carboxyglutamate protein (BGLAP), periostin (POSTN), osteopontin (OPN), osterix (OSX), collagen type I alpha 1 chain (COL1A1), dentin matrix acidic phosphoprotein (DMP)1, RUNX family transcription factor (RUNX)2, and marker of proliferation Ki-67 (KI67) was quantified by real-time PCR. Protein expression was analyzed by immunocytochemistry and ELISA. ALP activity was determined by ALP assay. Von Kossa and alizarin red staining were used to display mineralization. Cell viability was analyzed by XTT assay, and morphological characterization was performed by DAPI/phalloidin staining. Cell migration was quantified with an established scratch assay. CAP and EMD upregulated both mRNA and protein synthesis of ALP, POSTN, and OPN. Additionally, DMP1 and COL1A1 were upregulated at both gene and protein levels. In addition to upregulated RUNX2 mRNA levels, treated cells mineralized more intensively. Moreover, CAP treatment resulted in an upregulation of KI67, higher cell viability, and improved cell migration. Our study shows that CAP appears to have stimulatory effects on regeneration-associated cell functions in cementoblasts.

## 1. Introduction

Traumatic dental injuries (TDI) are common events in the dental field. It has been shown that approximately 900 million people in the world population of 2016 had at least one dental traumatic event [1]. Dental trauma includes fractures, luxations, and avulsion of primary and permanent teeth, with crown fractures and luxations being the most common [2]. When a tooth is injured, often anatomical structures such as cementum, periodontal ligament, alveolar bone, and gingiva are also damaged and require to regenerate.

Another reason for the pathological loss of these anatomical structures is periodontitis. Periodontitis is a multifactorial chronic inflammatory disease characterized by attachment and bone loss. About 10% of the world’s population is thought to be affected by severe periodontitis [3]. The pathological biofilm triggers and keeps periodontitis active, leading to progressive and irreversible loss of periodontal ligament, cementum, and alveolar bone [4]. Conventional treatment only results in periodontal repair; however, in certain circumstances, regenerative treatment approaches such as enamel matrix derivates (EMD) are possible [5,6].

Stem cells also play an important role in the oral cavity as part of regenerative processes and can be obtained, for example, from the pulp, the periodontium, or the apical follicle [7,8]. Stem cells interact closely with surrounding cells and are strongly influenced by the microenvironment. This includes, e.g., mechanical stimuli, but also biochemical ones [9]. Regenerative processes in the periodontium aim at changing the microenvironment in order to promote proliferation and differentiation of stem cells and, in turn, periodontal regeneration. For example, EMD has been shown to support the proliferation and differentiation of mesenchymal stem cells [10]. Especially the regeneration of cementoblasts is a difficult challenge concerning TDI and periodontitis [11]. Cementoblasts are covering the root surface and have similar molecular properties to osteoblasts, including the production of type I collagen, bone sialo-protein, osteocalcin, and osteopontin [11]. In contrast, however, dental cementum is not vascularized or innervated [12,13]. If periodontal ligament and cementum are damaged in case of TDI, there is a risk that the defect will be regenerated by osteoblasts, which may eventually lead to ankylosis and later tooth loss [14].

Many in vitro studies have shown the stimulating effects of EMD on periodontal ligament (PDL) cells, gingival fibroblasts, osteoblasts, and cementoblasts regarding cell regeneration [15,16,17,18,19]. An improvement in periodontal wound healing has also been observed in many clinical studies using EMD [20,21,22]. Additionally, a modulated production of cytokines regarding an anti-inflamed environment has also been observed, ensuring a less complicated healing process [23].

In recent years, cold atmospheric plasma (CAP) has become known as an interesting method for potentially accelerating wound healing processes [24,25,26]. CAP corresponds to a highly reactive gas, also known as the fourth state of matter, which can be produced by noble gases or by the ambient air [27,28,29]. It consists of reactive oxygen (ROS) and nitrogen species (RNS), ions, radicals, electric fields, and electromagnetic radiation [30,31]. In vitro studies have shown the stimulating effects of CAP on cell proliferation and cell migration [32]. Additionally, CAP has shown a strong antimicrobial effect and appears to have a comparatively selective effect on tumor cells in vitro [33,34,35,36,37]. Overall, the CAP-specific impact on eukaryotic cells is largely unknown.

Due to the frequency of TDI and the high prevalence of periodontitis, the need for rapid and effective therapy of damaged tissue, CAP offers an interesting application horizon for the regeneration of affected tissue. CAP could be applied to the root surface of an avulsed tooth prior to replantation but could also be used after subgingival instrumentation of the root surface of periodontally diseased teeth. In a recent study, we have shown the stimulating effect of CAP on periodontal ligament cells [38]. As the application of EMD is the gold standard in dental hard tissue regeneration therapy, the aim of the presented study was to determine the effects of CAP on cementoblasts and its potential for the mineralization and cell proliferation compared to EMD.

## 2. Results

### 2.1. Regulation of Crucial Cementoblast Genes in CAP-Treated OCCM-30 Cells

**Figure 1:**
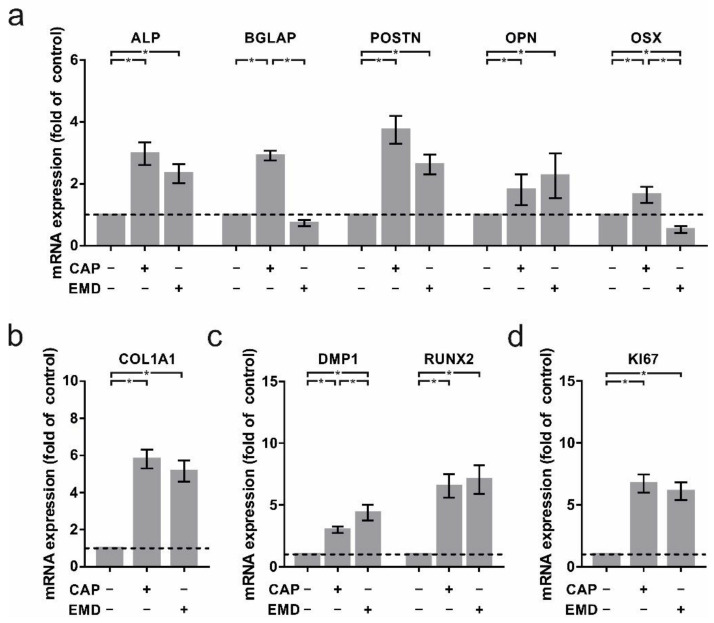
mRNA regulation of crucial markers in OCCM-30 cells after 60 s of CAP and 100 µg/mL of EMD treatment, respectively (+) as compared to untreated cells (−). (**a**) mRNA expression of ALP, BGLAP, POSTN, OPN, and OSX at one day (*n* = 15). (**b**) mRNA expression of COL1A1 at one day (*n* = 15). (**c**) mRNA expression of DMP1 and RUNX2 at one day (*n* = 15). (**d**) mRNA expression of KI67 at one day (*n* = 15). * statistical significance (*p* < 0.05).

CAP led to increased mRNA levels of crucial cementoblast- and mineralization-specific genes 24 h after treatment. ALP (3.0 ± 0.4-fold), BGLAP (2.9 ± 0.2-fold), POSTN (3.7 ± 0.5-fold), OPN (1.8 ± 0.1-fold), and OSX (1.6 ± 0.1-fold) were significantly upregulated compared to untreated controls. Remarkably, CAP-treated cells demonstrated similar results to EMD treatment, which was used as a positive control (ALP: 2.3 ± 0.3-fold; POSTN: 2.6 ± 0.3-fold; OPN: 2.3 ± 0.2-fold; Figure 1a). Interestingly, BGLAP (0.7 ± 0.1-fold) and OSX (0.5 ± 0.1-fold) gene regulation were downregulated after EMD treatment (Figure 1a). Especially COL1A1 mRNA induction was particularly high after both CAP (5.6 ± 0.5-fold) and EMD (5.2 ± 0.6-fold) treatment (Figure 1b). In addition, DMP1 and RUNX2, as regulators of mineralization, have also been shown to significantly induce mRNA levels. After 60 s CAP treatment, expression of both factors increased significantly (DMP1: 3.0 ± 0.3-fold; RUNX2: 6.5 ± 0.9-fold; Figure 1c) as well as after incubation with EMD (DMP1: 4.4 ± 0.6-fold; RUNX2: 7.1 ± 1.2-fold; Figure 1c). These effects were accompanied by an upregulation of the proliferation factor Ki-67 (Figure 1d). After CAP treatment (6.7 ± 0.7-fold) as well as after incubation with EMD (6.1 ± 0.7-fold), KI67 mRNA levels increased significantly.

### 2.2. Increased Regulation of Proteins Associated with Cementogenesis in OCCM-30 Cells

**Figure 2:**
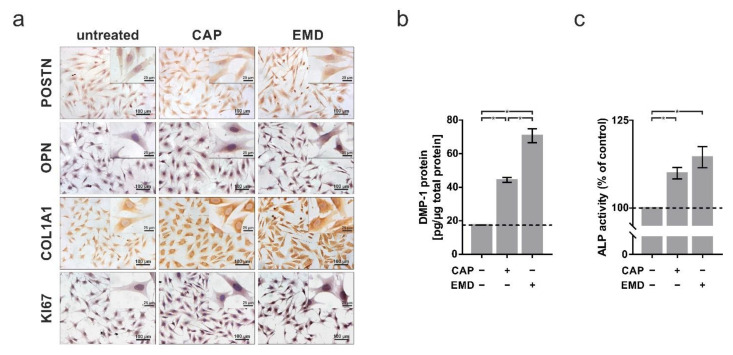
Protein regulation of selected markers in OCCM-30 cells after 60 s of CAP and 100 µg/mL of EMD treatment, respectively (+) as compared to untreated cells (−). (**a**) Immunocytochemistry staining of POSTN, OPN, COL1A1, and KI67 at two days. The scale bars represent 100 μm and 25 µm, respectively. (**b**) Protein expression of DMP1 normalized to total protein at two days (*n* = 9). (**c**) ALP activity at two days ALP activity in OCCM-30 cells after 60 s of CAP and 10 µg/mL of EMD at two days (*n* = 9). * statistical significance (*p* < 0.05).

These results could be confirmed at the protein level. Immunocytochemical analyses demonstrated 48 h after treatment that both CAP and EMD led to an increase in protein levels of POSTN (CAP: 129 ± 4.6%; EMD: 126 ± 3.6%), OPN (CAP: 119 ± 1.3%; EMD: 144 ± 3.1%), COLA1 (CAP: 148 ± 2.0%; EMD: 169 ± 6.4%), and KI67 (CAP: 130 ± 3.4%; EMD: 126 ± 3.6%) in the cells (Figure 2a). DMP1 protein was also significantly induced by both treatments (CAP: 44.3 ± 1.4 pg/µg of total protein; EMD: 70.7 ± 4.2 pg/µg of total protein; Figure 2b), as could be detected by ELISA in total lysates. To confirm the induction of essential factors, an ALP activity assay was performed. Measurement demonstrated induction of enzyme activity as a marker for physiological activity of the cells 48 h after treatment with CAP (109.9 ± 1.6%) and EMD (114.5 ± 3.0%; Figure 2c).

### 2.3. Accelerated Mineralization after Both CAP and EMD Treatment in OCCM-30 Cells

**Figure 3:**
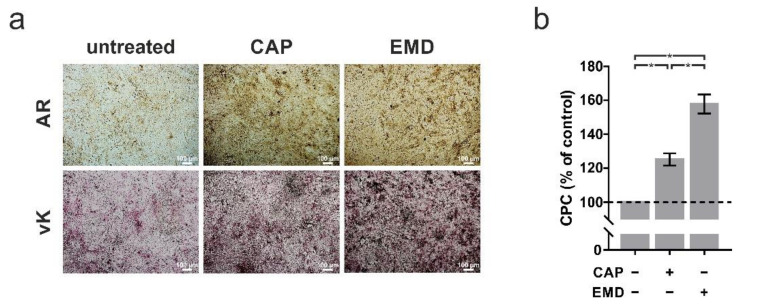
Effects of CAP and EMD application (100 µg/mL) mineralization in OCCM-30 cells (+) as compared to untreated cells (−). (**a**) Alizarin Red (AR) and von Kossa (vK) staining of OCCM-30 cells cultivated in medium for induction of mineralization at 7 days. The scale bars represent 100 μm. (**b**) Spectrophotometric quantification of AR staining, using the CPC extraction method (*n* = 9). * statistical significance (*p* < 0.05).

Beyond the physiological cell processes, we focused in the following on the mineralization capacity of cementoblasts as an important function of this cell type. A 60 s treatment with CAP every 2 d resulted in intensive mineralization after 7 d, as demonstrated by alizarin red (AR) and von Kossa (vK) staining (Figure 3a). Here, incubation with EMD resulted in significantly stronger mineralization than treatment with CAP, as confirmed by CPC quantification (Figure 3b). While CAP increased mineralization to 125%, EMD increased mineralization to 158%.

### 2.4. Higher Proliferation, Migration and Cell Viability after Both CAP and EMD Treatment in OCCM-30 Cells

**Figure 4:**
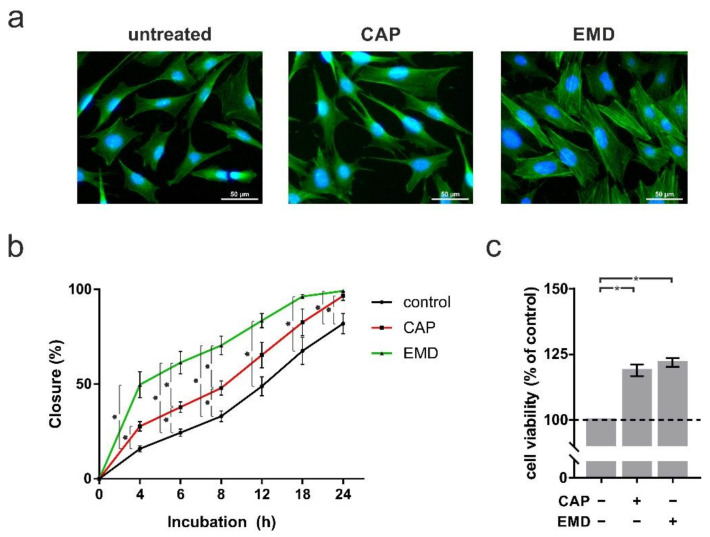
Analysis of proliferation and migration after 60 s of CAP and 100 µg/mL of EMD treatment, respectively, at one day (+) as compared to untreated cells (−). (**a**) Actin cytoskeleton of OCCM-30 cells. Cytoskeleton and nucleus are stained with FITC conjugated phalloidin (green) and DAPI (blue), respectively. The scale bars represent 50 μm. (**b**) Quantitative evaluation of scratch assay, displaying cell migration (*n* = 6). (**c**) cell viability visualized by XTT (*n* = 9). * statistical significance (*p* < 0.05).

Structural analyses indicated that CAP and EMD could also cause morphological and physiological changes in the cementoblasts. Staining of the actin component of the cytoskeleton with phalloidin revealed increased cell protrusions after both CAP and EMD treatment (Figure 4a). As this could suggest increased mobility, a scratch assay was performed to quantify cell motility. After CAP treatment, cementoblasts showed a moderate increase in mobility. In the presence of EMD, however, cementoblasts were clearly and statistically significantly more mobile than control cells (Figure 4b). This was accompanied by the increased physiological activity of the cementoblasts. An XTT viability assay showed that CAP, as well as EMD treatment of the cells, led to a significant increase in cell physiological activity. Interestingly, treatment with CAP (118.9% ± 2.2%) led to a similar increase in physiological activity at one day as treatment with EMD (121.9% ± 1.7%; Figure 4c). The section may be divided by subheadings. It should provide a concise and precise description of the experimental results, their interpretation, as well as the experimental conclusions that can be drawn.

## 3. Discussion

**Figure 5:**
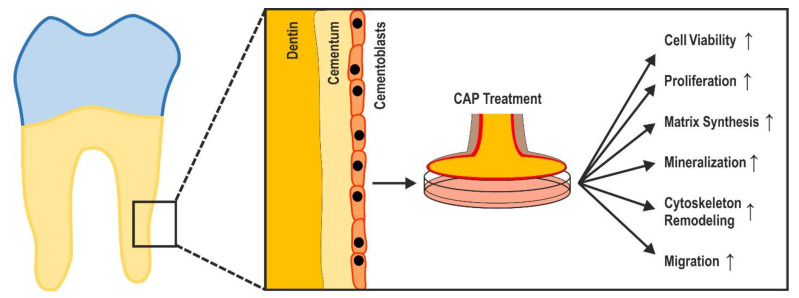
Markers of periodontal regeneration (cell viability, proliferation, matrix synthesis, mineralization, cytoskeleton remodeling, migration) were enhanced by CAP in murine dental cementoblasts.

In the present study, we have demonstrated the stimulating effect of CAP treatment on regeneration-associated cell functions and mineralization abilities of cementoblasts (Figure 5). To the best of our knowledge, our findings show, for the first time, similar effects of CAP and EMD, which are well known for EMD [16,39,40].

In our previous studies, we have addressed the influence of CAP on periodontal ligament cells and bone cells [38,41]. Since periodontal ligament cells can adopt both a cementoblast and an osteoblast phenotype and all of these cells play an important role in periodontal regenerative processes, in this study, we also wanted to investigate the effect of CAP on cementoblasts.

To characterize the formation of hard tissue in OCCM-30 cells after CAP treatment, we investigated the gene expression of the cementoblast- and mineralization-specific genes ALP, OPN, and POSTN.

ALP plays an important role in the initiation and induction of the first steps of cementum mineralization, in which primarily calcium and phosphorus are integrated into the developing teeth [42]. This maturation process is essential for resistant teeth that must withstand chewing forces and external influences [43]. Our results, which show higher ALP expression and activation levels suggest the ability of CAP to promote mineralization and thus tooth regeneration.

As a major component of the extracellular matrix of cementoblasts, the OPN protein also represents an important regulator of tooth mineralization and controls the deposition of hydroxyapatite [44,45]. As another factor, POSTN is also involved in tooth mineralization and tooth development in general [46,47]. POSTN also induces cell motility and recruitment and thus mediates another crucial function in tooth regeneration [47]. The upregulation of both OPN and POSTN after CAP treatment demonstrates the regenerative efficacy of CAP on cementoblasts at the molecular level.

As a further factor of the extracellular matrix, DMP1 is also responsible for the mineralization of dentin [48,49]. Our results showed that this protein is also induced by CAP and thus may also contribute to CAP-mediated mineralization of cementoblasts.

The collagen isoform collagen 1α is encoded by the gene COL1A1. It is characteristic of cementoblasts and constitutes the main part of the extracellular matrix of dental cementum [50,51]. The extracellular protein is upregulated both at mRNA and protein level after CAP treatment, indicating a CAP-dependent increase in the overall physiological activity of the cementoblasts.

The physiological activation of the cementoblasts has been confirmed by the upregulation of RUNX2. This protein is a key transcription factor and controls the cell differentiation of cementoblasts during initial tooth development [52,53]. CAP treatment also has induced the expression of RUNX2, indicating the maturation of cementoblasts and the promotion of tissue regeneration processes. Within 7 d there was a significant increase in cell mineralization, which is consistent with the effect of EMD [16]. In addition, longer incubations with inductive medium of 9 d or 10 d have been described in the literature [54,55]. Regarding therapeutic use, the CAP-mediated mineralization effect could possibly be further enhanced by longer treatment times, repeated treatments, or longer incubation times.

In particular, the markers BGLAP and OSX play a prominent role in the mineralization of cementoblasts. Both genes were significantly upregulated by CAP after one day. Interestingly, stimulation with EMD resulted in a downregulation of BGLAP and OSX mRNA expression in OCCM-30 cells after one day. This has also been shown by other authors in human gingival mesenchymal cells, dental follicle cells, and cementoblasts [56,57,58]. However, Wu et al. subsequently observed an increase in BGLAP mRNA expression after 3 d in human gingival mesenchymal cells [58]. In osteoblasts, an increase in BGLAP mRNA expression after stimulation with EMD has been shown at 14 d [59]. Other authors observed an increase in OSX mRNA at 7 d following EMD stimulation [60]. The discrepancy between the results may be due to differences in cell types, culture conditions, methods of EMD stimulation, or differences in EMD concentration. It is also possible that upregulation of BGLAP or OSX by EMD can only be observed after several days of incubation. Nevertheless, it is interesting that 60 s of CAP treatment already shows BGLAP upregulation at one day. Further studies on longer time points are necessary to clarify this point of difference. Currently, a number of other factors have been described that belong to the complex signal and effector network of cementoblast mineralization. In the literature, there are such diverse cellular factors as BMP2, BSP, TGFβ, and OPG [61,62,63], which may also be involved in or influence the CAP-induced process of mineralization.

In addition to the functional activation of cementoblasts, treatment with CAP also promoted cell proliferation. Both the physiological activity (XTT assay, cytoskeleton structure), as well as the expression of the proliferation marker Ki-67, exhibited a significant proliferative and thus regenerative effect of CAP treatment. This efficacy was also observed in other cell types such as keratinocytes and PDL cells [38,64,65] and is also used in dermatology for the treatment of chronic wounds [66]. In the latter application, CAP-induced cell mobility also plays a major role [32,38,67], which was also demonstrated in the present study. Both morphological changes and functional scratch assays demonstrated the motility-enhancing effect of CAP on cementoblasts. However, there are also conflicting reports that CAP can lead to suppression of Ki-67 [68] and to growth inhibition and apoptosis [65,69,70]. The biological effect of CAP treatment seems to depend significantly on both the cell type and the plasma source used [70]. This must be taken into account with regard to the therapeutic application, and plasma devices must be thoroughly evaluated for their biological properties before potential clinical use. Especially the technical differences between plasma devices (carrier gas, gas flow rate, device power, frequency of the high-frequency generator) hamper a systematic comparison of such examinations [71].

Interestingly, it has been shown that stem cells interact closely with the surrounding cells and are strongly influenced by the microenvironment [9]. It has been shown that CAP both promotes the formation of ROS and RNS and induces electric fields and electromagnetic radiation [30,31]. In this respect, it could be that CAP positively influences the microenvironment of periodontal stem cells through these mechanisms with the effect of promoting periodontal regeneration.

In studies on the CAP effect, no defined condition of CAP treatment can be specified that reproducibly leads to a defined biological effect. For EMD, as for pharmacological agents, a concentration can be defined, and dose-dependent effects can be detected [72,73]. In CAP treatment, it is not possible to define a concentration (of reactive species, for example) because these species continue to react very rapidly and have very short half-lives [74]. The only practical specification is the treatment duration, which leads to reproducible treatment duration-dependent effects [38,75]. In the present study, only a 60 s treatment was performed. Further systematic investigations with CAP treatments of different durations are necessary to characterize CAP effects on cementoblasts more precisely. This could also help to capture the differences between CAP and EMD treatment more accurately. Furthermore, a combination of CAP and EMD would also be a feasible approach that could potentially lead to synergistic effects.

In the present study, we analyzed DMP-1 and ALP as components of the secretome. Due to the numerous other analyses (viability, gene expression, mineralization, morphology, migration, etc.), we restricted ourselves to these two secretome components. Future studies should clarify how other components of the secretome, including the matrisome, are regulated by CAP. Another limitation of our study is that we did not examine the combined effect, i.e., the combination of CAP and EMD on cementoblasts. In the present study, EMD served only as a regenerative control agent. Future studies will have to show whether the combination of CAP and EMD leads to additive or synergistic effects. Furthermore, in the present study, we only investigated the effect of CAP at one distance length and applied it once for 60 s. Further studies need to clarify whether the observed effects also occur at other distances and longer and multiple applications. Furthermore, murine cells, which were immortalized, were used in our experiments. Future studies should, therefore, also be performed with primary human cementoblasts.

Furthermore, it should be noted that murine cementoblasts are probably not fully comparable to human primary cells so that investigations on primary human material are mandatory before patient studies.

With regard to clinical use, potential side effects, which have been described for direct application of CAP on the oral mucosa, must be ruled out [76]. Additionally, it should be considered that after replantation of a tooth, cementum cells are no longer directly accessible for CAP treatment. Therefore, only the application before replantation of the tooth would be possible. However, fluids such as buffer solutions or cell media can also be treated with CAP and indirectly mediate the biological effects. Although these are usually somewhat less pronounced, they allow further application options such as flushing [77]. This could allow application in other dental traumas involving injury of the cementum, such as luxations or root fractures. Our results show that CAP promotes cementoblast properties associated with regeneration. Our results, therefore, suggest that the application of CAP to an avulsed tooth may promote healing after its reimplantation. Furthermore, the application of CAP after subgingival instrumentation of the root surface of periodontally diseased teeth could be beneficial for reparative or, ideally, even regenerative healing. Before clinical use, both types of CAP application need to be clarified in the animal model.

## 4. Materials and Methods

### 4.1. Cell Culture

Immortalized mouse cementoblast cells OCCM-30 were used for this study. Cell isolation and immortalization method have been described previously [51,78]. Cells were kindly provided by Dr. Martha J. Somerman (NIDCR, NIH, MD, USA) and stored at −80 °C. Cells were propagated in Dulbecco’s modified essential medium (DMEM, Invitrogen, Waltham, MA, USA) supplemented with 10% fetal bovine serum (FBS, Invitrogen), 100 units penicillin, and 100 μg/mL streptomycin (Invitrogen) in 75 cm^2^ cell culture flasks (Greiner Bio-One, Kremsmünster, Austria) at 37 °C with 5% CO_2_ and 95% humidity. The cell culture medium was replaced every two days. For experiments, cells were seeded at 2.5 × 10^4^ viable cells/mL into 3.5 cm cell culture Petri dishes (VWR, Radnor, PA, USA) and cultured to 70% confluence. One day prior to the experiments, FBS concentration was reduced to 1%. For long-term experiments, cells were CAP-treated every second day, immediately after replacing of culturing medium.

### 4.2. CAP Treatment

CAP was generated by a dielectric barrier discharge with ambient air as carrier gas (Plasma ONE MEDICAL, Plasma MEDICAL SYSTEMS^®^ GmbH, Nievern, Germany) with an output of 18 kV. Cells were exposed to CAP for 60 s as previously described [38]. EMD (Straumann AG, Basel, Switzerland) served as a positive control for mineralization at various concentrations (0.01/0.1 mg/mL). Untreated cells served as a negative control.

### 4.3. Analysis of Gene Expression

For transcriptional analysis, total RNA was isolated using an RNA extraction kit (RNeasy Protect Minikit, Qiagen, Hilden, Germany) in accordance with the manufacturer’s instructions. RNA concentration was measured using a NanoDrop ND-2000 spectrophotometer (Thermo Fisher Scientific, Wilmington, DE, USA). Subsequently, 1 μg RNA was reverse transcribed into cDNA using iScript™ Select cDNA Synthesis Kit (Bio-Rad Laboratories, Munich, Germany) at 42 °C for 90 min according to the manufacturer’s instructions. The expression of the target mRNA was detected by real-time PCR with 1 µL cDNA in a reaction mixture of 25 µL containing 2.5 μL specific commercially available pre-designed primers (QuantiTect Primer assay, Qiagen), 12.5 μL SYBR Green QPCR Master Mix (Bio-Rad), and 9 μL deionized water. The following protocol was used for amplification: Initial denaturation and thermal activation of the polymerase (95 °C, 5 min), followed by 40 cycles of denaturation (95 °C, 10 s), and a combined annealing/elongation (60 °C, 30 s). mRNA levels of ALP, BGLAP, POSTN, OPN, OSX, COL1A1, DMP1, RUNX2, and KI67 detected by real-time PCR using the iCycler iQ™5 detection system (Bio-Rad). Data were analyzed by the comparative threshold cycle method using glyceraldehyde-3-phosphate dehydrogenase (GAPDH) as a reference gene.

### 4.4. Immunocytochemistry

OCCM-30 cells were seeded on glass coverslips (Thermanox) and treated as described above. After one and two days, cells were fixed with 4% paraformaldehyde (Merck KGaA, Darmstadt, Germany) for 10 min and permeabilized with 0.05% Triton X-100 (Merck) in PBS. Blocking of non-specific binding was performed with 5% bovine serum albumin faction V (Roche Diagnostics, Indianapolis, IN, USA) in 1× PBS for 45 min at room temperature. Primary antibodies were incubated for 1,5 h at room temperature or overnight at 4 °C: rabbit polyconal anti-collagen I antibody (1:200; Abcam, Cambridge, UK), rabbit polyclonal anti-osteoblast specific factor 2 antibody (1:1000; BioVendor, Brno, Czech Republic), rabbit anti-osteopontin (1:200; Abcam), and rabbit anti-Ki67 antibody (1:200; Abcam). Peroxidase-conjugated anti-mouse or anti-rabbit EnVision^®^ (Dako, Glostrup, Denmark) were used as secondary antibodies for 30 min at room temperature and visualized with diaminobenzidine (DAB). OCCM-30 cells were counterstained with hemalaunum (Merck). Semi-quantitative analysis of stained cells was performed using ImageJ Fiji (ImageJ 2.1.0/1.53c, National Institutes of Health, Bethesda, MD, USA) as described in the literature [79]. In each case, 3 sections of 3 independent experiments were randomly selected for measurement.

### 4.5. Analysis of Protein Levels

For quantification of protein levels of DMP-1, OCCM-30 cell supernatants were analyzed by the Mouse DMP-1 enzyme-linked immuno assay (ELISA) kit (Abcam) according to the manufacturer’s instructions. Absorbance was measured with a microplate reader (Epoch™ Microplate Spectrophotometer, BioTek Instruments, Winooski, VT, USA) at 450 nm.

### 4.6. ALP Assay

To measure ALP enzyme activity, cells were washed twice with cold PBS one or two days after CAP treatment, lysed with 0.05% Triton X-100 (Merck), scraped, and analyzed with an ALP activity kit (Merck). In order to normalize ALP-specific changes in OCCM-30 cell number, the protein content of each sample was determined by a BCA protein assay kit (Thermo Fisher Scientific). The absorbance was measured with a microplate reader as described above at 405 nm and 570 nm, respectively.

### 4.7. Von Kossa Staining

For evaluation of mineralization in OCCM-30 cell culture, von Kossa staining was performed. Cells were cultivated on coverslips and treated with CAP and EMD, respectively, for 1 d, 2 d, and 7 d. At the end of incubation, cells were washed twice with PBS and fixed with 4% paraformaldehyde (Merck) for 10 min at room temperature. After rinsing with deionized water, cells were treated with 5% silver nitrate solution (Merck) for 40 min at 4 °C. After another washing step with deionized water, cells were incubated with 1% pyrogallol (Merck) for 5 min at room temperature. Following rinsing with deionized water, unreacted silver was removed with the incubation of sodium thiosulphate (Merck) for 5 min at room temperature. After a final washing step with deionized water, cells were counterstained for 10 min at room temperature with 0.1% nuclear fast red solution. The stained preparations were rinsed, then dehydrated 2 × 2 min by 100% ethanol (Merck) and 2 × 2 min by xylene, and finally overlaid with DePeX (Serva Feinbiochemica, Heidelberg, Germany). Prepared cells were analyzed by light microscopy at 5-fold magnification (Axioskop 2, Axiocam MRc, Axiovision 4.7/AutMess, Zeiss, Oberkochen, Germany). For each group, three sections of three independent experiments were randomly selected for the measurements.

### 4.8. Alizarin Red Staining

Alizarin red S (Merck) staining and cetylpyridinium chloride (CPC; Merck) was used to analyze the accumulation of calcium in OCCM-30 cells, which has previously been described by Reinholz et al. 2000 [80]. Cells were cultivated for 7 d and fixed in paraformaldehyde (Merck) at RT for 10 min. Subsequently, cells were washed twice with PBS and permeabilized with 0.05% Triton X-100 (Merck) in PBS for 5 min. After washing twice with PBS, the cell monolayer was then incubated with 500 µL alizarin red S (40 mM, pH 4.2) for 20 min at RT. Afterward, the cells were washed five times with 500 µL dH20. Stained OCCM-30 cells were analyzed by light microscopy as described above. To quantify mineralization, the stained monolayer was incubated with 10% CPC in 10 mM Na_2_PO_4_ (pH 7.0) for 20 min. Finally, the cell monolayer was completely detached from the surface of the well, resuspended, and the cell suspension was centrifuged at 20,000× *g* for 10 min at RT. The supernatant was analyzed with a microplate reader as described above at an absorbance of 562 nm.

### 4.9. Analysis of Cell Morphology

For immunofluorescence staining, OCCM-30 cells were seeded on coverslips and cultured to 70% of confluence. One day prior to the experiments, serum concentration was reduced as described above. Cell morphology was analyzed with a phalloidin/DAPI staining after fixation and permeabilization of the cells. A total of 100 µM phalloidin (Merck) was used for 40 min in order to label the actin filaments. After washing, 1 µg/mL DAPI (Merck) was applied for 5 min to label DNA. Stained cells were analyzed with the ZOE™ Fluorescent Cell Imager (Bio-Rad) as described above.

### 4.10. Scratch Assay

The scratch assay was performed in order to assess cell motility of OCCM-30 cells after CAP and EMD treatment, respectively, compared to untreated cells. The confluent cell monolayer was scratched by using a standardized sterile instrument following a previously described protocol [81]. The confluence of the monolayer comprising a 3 mm cell-free area was quantified every 15 min for 24 h using time-lapse image capturing, which was performed automatically by the integrated camera of the device (JuLI™ Br and JuLI™Br PC software, NanoEnTek, Seoul, Korea).

### 4.11. XTT Assay

The XTT cell viability kit (Cell Signaling Technology, Danvers, MA, USA) was used to study the physiological activity of cells. Cells were treated with CAP and EMD, respectively, and incubated for 24 h as described above. Subsequently, XTT reaction solution was added to the culturing medium according to the manufacturer’s instructions. Absorbance was determined after 4 h of incubation with a microplate reader as described above at 475 nm.

### 4.12. Statistical Analysis

Experiments were performed in triplicates and repeated at least three times by calculating mean values and standard errors of the mean (SEM). GraphPad Prism Software (GraphPad Software, San Diego, CA, USA) was used for statistical analysis, and Kruskal–Wallis test and Mann–Whitney U test with Bonferroni–Holm correction were performed at the significance level of *p* < 0.05.

## 5. Conclusions

In conclusion, our data show that treatment with CAP can stimulate regeneration-associated processes in dental cementoblasts. This property makes CAP another promising treatment option for TDI and may potentially supplement classical treatment with EMD.

## Data Availability

Data sharing is not applicable to this article.
